# Synthesis of zirconium(iv) and hafnium(iv) isopropoxide, *sec*-butoxide and *tert*-butoxide†

**DOI:** 10.1039/d4dt01280a

**Published:** 2024-06-28

**Authors:** Evert Dhaene, Carlotta Seno, Jonathan De Roo

**Affiliations:** a Department of Chemistry, University of Basel Mattenstrasse 22 4058 Basel Switzerland Jonathan.DeRoo@unibas.ch

## Abstract

We revisited the synthesis of zirconium(iv) and hafnium(iv) alkoxides, namely the metal isopropoxide isopropanol complex (M(OiPr)_4_·iPrOH, M = Zr, Hf) and the metal *sec*- and *tert*-butoxide (M(O*s*Bu)_4_ and M(O*t*Bu)_4_, M = Zr, Hf). We optimized the most convenient synthesis methods and compared the products with commercial sources. En route to the metal *sec*- and *tert*-butoxides, we synthesized the metal diethylamido complex (M(NEt_2_)_4_, M = Zr, Hf).

## Abstract 2

We revisited the synthesis of zirconium(iv) and hafnium(iv) alkoxides, namely the metal isopropoxide isopropanol complex (M(OiPr)_4_·iPrOH, M = Zr, Hf) and the metal *sec*- and *tert*-butoxide (M(O*s*Bu)_4_ and M(O*t*Bu)_4_, M = Zr, Hf).

## Graphical abstract

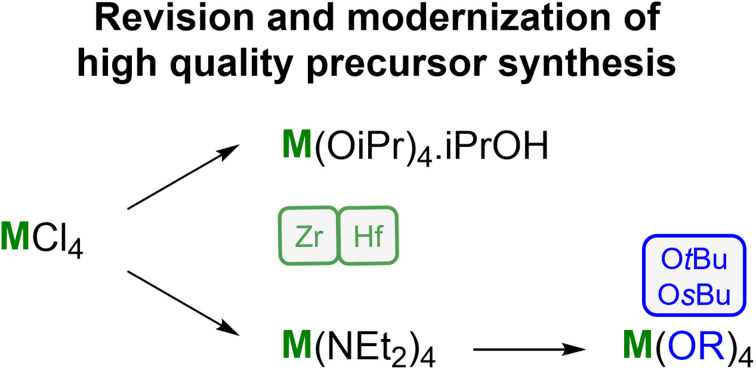

## Introduction

Zirconium and hafnium alkoxides are important reagents in the production of various materials, in particular for (doped) oxide nanocrystals (NCs).^1–25^ Specific examples are1
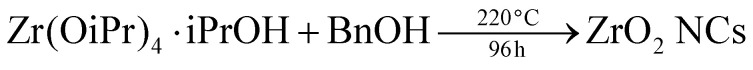
2
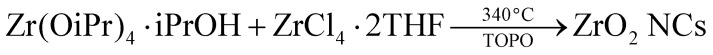
3
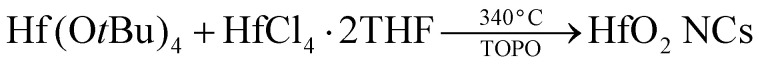
where BnOH is benzyl alcohol, THF is tetrahydrofuran and TOPO is tri-*n*-octylphosphine oxide. In the surfactant-assisted synthesis in TOPO, the size of oxide nanocrystals can be tuned by varying the metal alkoxide,^19,23^ for example, replacing zirconium isopropoxide with zirconium *tert*-butoxide reduces the diameter from 4 to 3 nm.^19^

In our laboratory, we have ample experience in producing oxide nanocrystals and oxo clusters from metal alkoxide precursors.^1,3,18,19,24–30^ While zirconium and hafnium isopropoxide and *tert*-butoxide compounds are commercially available, we experienced a high supplier-to-supplier and batch-to-batch variability in terms of color of the isopropoxide (from slightly yellow to brown instead of white) or purity of the *tert*-butoxide (*e.g.*, turbid instead of transparent). Given that reagent purity directly affects the reproducibility of chemical reactions, this is cause for concern. It is advised to purify these commercial precursors by means of a recrystallization or vacuum distillation.^31^ In addition, such specialty chemicals are often back-ordered, and long delivery times are normal.

To secure a reliable supply of high-quality precursors, we sought to synthesize the metal alkoxides in our laboratory, thus also gaining access to commercially unavailable precursors, such as zirconium *sec*-butoxide. To achieve this, we revisited synthetic methods published in the previous century, and we provide here modernized synthetic protocols with an elaborate description to make them widely accessible. Note that zirconium and hafnium alkoxides are moisture sensitive and thus rigorous anhydrous conditions are required for synthesis and storage.

## Experimental

### Materials

All manipulations were performed under rigorously anhydrous conditions *via* dry nitrogen or argon atmosphere and standard Schlenk and glovebox techniques unless otherwise mentioned. All chemicals were used as received unless otherwise mentioned. Hafnium(iv) chloride (99.9%), tri-*n*-octylphosphine oxide (99%), and zirconium(iv) chloride (99.5%) were purchased form Strem Chemicals. Ammonia (2 M in isopropanol), ammonia (gaseous), lithium diethylamide (95%), and anhydrous *tert*-butanol (99.5%) were purchased from Sigma-Aldrich. 2-Butanol (extra dry over molecular sieves), isopropanol (extra dry over molecular sieves), and toluene (extra dry over molecular sieves) were purchased from Thermo Scientific. Benzene-d_6_ (99.5 atom%D) is purchased from Apollo Scientific. Toluene was dried over a solvent system before being transferred into a glovebox. To remove residual water from solvents, 10% m/v of activated molecular sieves were added and left to stand for 3 days in the glovebox prior to use, which yield less than 1 ppm water as determined previously.^32^ Tri-*n*-octylphosphine oxide (TOPO) was recrystallized according to Owen *et al.*^33^

### Zr(OiPr)_4_·iPrOH complex synthesis from NH_3_ stock solution in isopropanol

The protocol is inspired by the reports of Bradley *et al.* and Seisenbaeva *et al.*^31,34,35^ In a nitrogen filled glovebox, a 500 mL Schlenk flask was loaded with zirconium(iv) chloride (14.0 g, 60 mmol, 1.0 eq.) and toluene (150 mL), and air- and moisture-free transferred to the Schlenk line. The ammonia solution in isopropanol (2 M, 135 mL, 270 mmol, 4.5 eq.) was slowly and carefully dropwise added to the zirconium suspension while cooling the Schlenk flask with a water bath since the reaction is slighlty exothermic. After 1 hour of stirring at room temperature, the formed ammonium chloride was removed by means of Schlenk filtration (500 mL frit, porosity 4 (10–16 μm)) to another 500 mL Schlenk flask, as depicted in Fig. S1.† The solvent was removed under reduced pressure, and the product was redissolved in toluene (100 mL) to which ammonia solution in isopropanol (2 M, 30 mL, 60 mmol, 1.0 eq.) was added dropwise. If the previous exchange was incomplete, additional ammonium chloride precipitates. After 1 hour of stirring at room temperature, the additional ammonium chloride was removed by Schlenk filtration (250 mL frit, porosity 4 (10–16 μm)) to a 250 mL Schlenk flask. The solvent was removed under reduced pressure. The resulting product was recrystallized to purity from a concentrated hot mixture (15 to 20 mL) of toluene and isopropanol (3 : 1 ratio by volume) where the hot solution was allowed to slowly crystallize overnight into colourless large crystals. The following day, the solvent was removed *via* Cannula transfer. If needed a second recrystallization could be done under the same conditions. The product was vacuum dried at room temperature (at higher temperatures, the coordinated isopropanol can be removed under reduced pressure) to obtain a white crystalline product (15.1 g, 65%) and is stored in the glovebox. No residual chloride was detected in the final product (*i.e.* <0.85% chloride to metal). ^1^H NMR (500 MHz, C_6_D_6_): *δ* 6.0–5.5 (s, 1H), *δ* 5.5–4.0 (s, 5H), *δ* 2.0–1.0 (s, 30H). ^13^C NMR (125 MHz, C_6_D_6_): *δ* 69.7, 26.6. SC-XRD confirmed the identity of the product as reported by Vaartstra *et al.* (CSD ID: JETWOU).^36^

### Hf(OiPr)_4_·iPrOH complex synthesis from NH_3_ stock solution in isopropanol

Hafnium(iv) isopropoxide isopropanol complex was synthesized similar to Zr(OiPr)_4_·iPrOH complex. Hafnium(iv) chloride (19.2 g, 60 mmol, 1.0 eq.) was used instead of zirconium(iv) chloride. After recrystallization a white crystalline solid is obtained (14.4 g, 51%) and stored in the glovebox. No residual chloride was detected in the final product (*i.e.* <0.85% chloride to metal). ^1^H NMR (500 MHz, C_6_D_6_): *δ* 7.5–7.0 (s, 1H), *δ* 5.5–4.0 (s, 5H), *δ* 2.0–1.0 (s, 30H). ^13^C NMR (125 MHz, C_6_D_6_): *δ* 69.7, 26.7. SC-XRD confirmed the identity of the product as reported by Veith *et al.* (CSD ID: NAYDAS).^37^

### Zr(OiPr)_4_·iPrOH complex synthesis from gaseous NH_3_

The protocol is inspired by the reports of Bradley *et al.* and Seisenbaeva *et al.*^31,34,35^ In a nitrogen filled glovebox, a 500 mL Schlenk flask was loaded with zirconium(iv) chloride (14.0 g, 60 mmol, 1.0 eq.) and toluene (150 mL), and air- and moisture-free transferred to the Schlenk line. While the suspension was cooled with an ice bath, the gaseous ammonia was let to bubble through it with a flow of 50 L h^−1^ by sticking the needle in the suspension, to which anhydrous isopropanol (28.9 g, 36.7 mL, 480 mmol, 8 eq.) was added dropwise. Here, an ice bath was used instead of a water bath since the reaction is much more exothermic. The ammonia gas was allowed to bubble through the solution for a total of 13 minutes (which equals a total addition 10.75 L, 480 mmol, 8 eq.), but cannot be stopped before all the isopropanol was added. After 1 hour of stirring at room temperature, the formed ammonium chloride was removed by means of Schlenk filtration (250 mL frit, porosity 4 (10–16 μm)) to another 250 mL Schlenk flask, as depicted in Fig. S1.† The solvent was removed under reduced pressure. The resulting product was recrystallized to purity from a concentrated hot mixture (15 to 20 mL) of toluene and isopropanol (3 : 1 ratio by volume) where the hot solution was allowed to slowly crystallize overnight into colourless large crystals. The following day, the solvent was removed *via* Cannula transfer. If needed a second recrystallization could be done under the same conditions. The product was vacuum dried at room temperature (at higher temperatures, the coordinated isopropanol can be removed under reduced pressure) to obtain a white crystalline product (14.2 g, 61%) and is stored in the glovebox. No residual chloride was detected in the final product (*i.e.* <0.85% chloride to metal). ^1^H NMR (500 MHz, C_6_D_6_): *δ* 6.0–5.5 (s, 1H), *δ* 5.5–4.0 (s, 5H), *δ* 2.0–1.0 (s, 30H). ^13^C NMR (125 MHz, C_6_D_6_): *δ* 69.7, 26.6. SC-XRD confirmed the identity of the product as reported by Vaartstra *et al.* (CSD ID: JETWOU).^36^

### Hf(OiPr)_4_·iPrOH complex synthesis from gaseous NH_3_

Hafnium(iv) isopropoxide isopropanol complex was synthesized similar to Zr(OiPr)_4_·iPrOH complex. Hafnium(iv) chloride (19.2 g, 60 mmol, 1.0 eq.) was used instead of zirconium(iv) chloride. After recrystallization a white crystalline solid is obtained (14.2 g, 50%) and stored in the glovebox. No residual chloride was detected in the final product (*i.e.* <0.85% chloride to metal). ^1^H NMR (500 MHz, C_6_D_6_): *δ* 7.5–7.0 (s, 1H), *δ* 5.5–4.0 (s, 5H), *δ* 2.0–1.0 (s, 30H). ^13^C NMR (125 MHz, C_6_D_6_): *δ* 69.7, 26.7. SC-XRD confirmed the identity of the product as reported by Veith *et al.* (CSD ID: NAYDAS).^37^

### Zr(NEt_2_)_4_ synthesis

Tetrakis(diethylamido)zirconium(iv) complex was synthesized based on reports of Diamond *et al.*, Kim *et al.*, and Bradley *et al.*^38–40^ In a nitrogen filled glovebox, a 250 mL round bottom flask was loaded with lithium diethylamide (8.70 g, 110 mmol, 4.23 eq.) and toluene (80 mL), to which zirconium(iv) chloride (6.06 g, 26 mmol, 1.00 eq.) was carefully and slowly added while stirring since the reaction is exothermic. After the addition, the flask was sealed with a septum and left stirring overnight at room temperature. The next day, the reaction mixture was filtered making use of a standard glass frit (porosity 4 (10–16 μm)) in the glovebox to remove the insoluble salts, and transferred to a 100 mL Schlenk flask to evaporate the solvent. Afterwards, the Schlenk flask was transferred air- and moisture-free to the Schlenk line on which a predried vacuum distillation set-up was mounted. During the distillation, the receiver Strauss flask was cooled with an ice bath. The pure product distilled over at 130 °C at 50 mTorr and is a colourless and transparent liquid product (6.6 g, 67%). It is stored in the freezer of the glovebox at −29 °C. No residual chloride was detected in the final product (*i.e.* <0.85% chloride to metal). ^1^H NMR (500 MHz, C_6_D_6_): *δ* 3.36 (quad, *J* = 7.0 Hz, 2H), *δ* 1.16 (t, *J* = 7.0 Hz, 3H). ^13^C NMR (125 MHz, C_6_D_6_): *δ* 43.6, 16.3.

### Hf(NEt_2_)_4_ synthesis

Tetrakis(diethylamido)hafnium(iv) complex was synthesized similar to Zr(NEt_2_)_4_. Hafnium(iv) chloride (8.33 g, 26 mmol, 1.00 eq.) was used instead of zirconium(iv) chloride. During the distillation, the receiver Strauss flask was cooled with an ice bath. The pure product distilled over at 145 °C at 50 mTorr and is a colourless and transparent liquid product (9.3 g, 76%). It is stored in the freezer of the glovebox at −29 °C. No residual chloride was detected in the final product (*i.e.* <0.85% chloride to metal). ^1^H NMR (500 MHz, C_6_D_6_): *δ* 3.37 (quad, *J* = 7.0 Hz, 2H), *δ* 1.16 (t, *J* = 7.0 Hz, 3H). ^13^C NMR (125 MHz, C_6_D_6_): *δ* 42.9, 16.3.

### Zr(O*t*Bu)_4_ synthesis

Zirconium(iv) *tert*-butoxide was synthesized according to the procedure of Thomas *et al.*^41^ In a nitrogen filled glovebox, a 100 mL Schlenk flask was loaded with tetrakis(diethylamido)zirconium(iv) (3.80 g, 3.70 mL, 10 mmol, 1 eq.) and toluene (40 mL), and a 25 mL Schlenk flask was loaded with *tert*-butanol (5.93 g, 7.65 mL, 80 mmol, 8 eq.) and toluene (10 mL). Both Schlenk flasks were transferred air- and moisture-free to the Schlenk line, where the zirconium containing flask was cooled with an ice bath. The *tert*-butanol solution was added dropwise to the zirconium suspension *via* Cannula transfer. Next, the ice bath was removed and the solution was allowed to heat to room temperature, and stirred for 1.5 hours. Afterwards, the solvent was removed under reduced pressure prior to vacuum distillation. During the distillation, the receiver Strauss flask was cooled with an ice bath. The pure product distilled over at around 45 °C at 50 mTorr and is a colourless and transparent liquid product (2.7 g, 71%). It is stored in the freezer of the glovebox at −29 °C. No residual chloride was detected in the final product (*i.e.* <0.85% chloride to metal). ^1^H NMR (500 MHz, C_6_D_6_): *δ* 1.32 (s, 36H). ^13^C NMR (125 MHz, C_6_D_6_): *δ* 75.3, 33.1.

### Hf(O*t*Bu)_4_ synthesis

Hafnium(iv) *tert*-butoxide was synthesized similar to Zr(O*t*Bu)_4_. Tetrakis(diethylamido)hafnium(iv) (4.67 g, 3.74 mL, 10 mmol, 1 eq.) was used instead of tetrakis(diethylamido)zirconium(iv). During the vacuum distillation, the receiver Strauss flask was cooled with an ice bath. The pure product distilled over at around 30 °C at 50 mTorr and is a colourless and transparent liquid product (2.2 g, 46%) and is stored in the freezer of the glovebox at −29 °C. No residual chloride was detected in the final product (*i.e.* <0.85% chloride to metal). ^1^H NMR (500 MHz, C_6_D_6_): *δ* 1.33 (s, 36H). ^13^C NMR (125 MHz, C_6_D_6_): *δ* 75.5, 33.2.

### Zr(O*s*Bu)_4_ synthesis

Zirconium(iv) *sec*-butoxide was synthesized similar to Zr(O*t*Bu)_4_. 2-Butanol (5.93 g, 7.34 mL, 80 mmol, 8 eq.) was used instead of *tert*-butanol. During the vacuum distillation, the receiver Strauss flask was not cooled with an ice bath since the viscosity increase of the product would obstruct the receiving flask. The pure product distilled over at around 170 °C at 50 mTorr the colourless and is a transparent but very viscous liquid product (2.2 g, 57%). It is stored in the glovebox. No residual chloride was detected in the final product (*i.e.* <0.85% chloride to metal). ^1^H NMR (500 MHz, C_6_D_6_): *δ* 4.39 (sext, *J* = 7.5 Hz, 4H), 1.99 (sept, *J* = 7.4 Hz, 4H), 1.79 (sept, *J* = 6.2 Hz, 4H), 1.48 (d, *J* = 6.1 Hz, 12H), 1.03 (m, *J* = 7.5 Hz, 12H). ^13^C NMR (125 MHz, C_6_D_6_): *δ* 77.2, 33.3, 23.5, 11.0.

### Quantification of chloride

To test the residual chloride content in the alkoxide and amido complexes, the Spectroquant chloride test protocol (1.14897.0001) for the range of 2.5 to 25 mg L^−1^ chloride is used. With this test, chloride ions react with mercury(ii) thiocyanate to form slightly dissociated mercury(ii) chloride. The thiocyanate released in the process in turn reacts with iron(iii) ions to form red iron(iii) thiocyanate that is determined photometrically. For this, 0.05 mmol of the product was loaded into a 4 mL vial, dissolved in 1 mL of tetrahydrofuran (THF), and taken out of the glovebox. The solution was added dropwise into aqueous ammonia (2 mL, 0.5 M) and left for 1 hour. The solution was acidified by adding aqueous nitric acid (3 mL, 0.5 M) to it. To separate the solid from the liquid phase, the mixture was centrifuged (10 min, 10k rpm). Afterwards, 5 mL of the clear solution was taken to which reagent I (2.5 mL) and reagent II (0.50 mL) were added, and left stirring for 1 min. A 10 mm cuvette was filled with the solution and immediately measured with UV-vis spectrometer. A blank sample was prepared identical without any product which was used as a baseline for the measurements. To validate the test, ammonium chloride (32.1 mg, 0.6 mmol) was dissolved in THF (10 mL), from which 1 mL is taken and prepared identically for the measurement. We obtained a value of 97.4% chloride to ammonium for the control, providing confidence in the method. To calculate the chloride concentration, the absorption at 445 nm is used: *c* = 28.2 × *A* (in mg L^−1^) with a maximum accuracy of the measurement value of ±1.2 mg L^−1^. The range of 2.5 to 25 mg L^−1^ chloride correlates with a range of 0.85 to 8.48% Cl to the amount of metal.

### NMR spectroscopy

Nuclear magnetic resonance (NMR) spectra were recorded at 298.15 K on a Bruker UltraShield 500 spectrometer operating at a ^1^H frequency of 500.13 MHz. Regular ^1^H, ^13^C, and ^31^P NMR spectra were acquired using the standard pulse sequences with a 30 degree pulse with a recycle delay of 1.5, 1.0, and 1.0 second from the Bruker library; zg30, zgpg30, zgpg30 respectively. ^13^C and ^31^P NMR spectra were acquired using inverse gated decoupling, and processed with a line broadening of 1 and 5 Hz to reduce noise respectively. All resonances are background-corrected. Chemical shifts (*δ*) are given in parts per million (ppm), and the residual solvent peak was used as an internal standard (C_6_D_6_: *δ* H = 7.16 ppm, *δ* C = 128.06 ppm). The signal multiplicity is denoted as follows: s (singlet), d (doublet), t (triplet), quad (quadruplet), quin (quintet), sext (sextet), sept (septet), and m (multiplet). Coupling constants are reported in hertz (Hz).

### UV-vis spectroscopy

Ultraviolet–visible (UV-vis) absorption spectra were recorded on a PerkinElmer Lambda 365.

### Powder XRD

Powder X-ray diffraction patterns were collected at room temperature in transmission mode using a Stoe Stadi P diffractometer with a micro-focused Cu-Kα-source (*λ* = 1.542 Å) equipped with a DECTRIS MYTHEN 1K detector. Measurements were conducted in the *θ*/2*θ* range of 5 Å–65 Å.

### Single crystal XRD

Single crystal X-ray powder diffraction (SC-XRD) data were collected on STOE STADIVARI diffractometer with a microfocused Cu source. The crystals were kept at a steady temperature of 150 K during data collection.

## Results and discussion

Different synthetic routes have been reported for zirconium and hafnium alkoxides. The electrochemical route involves the dissolution of a metal anode in alcohol.^42^ The reaction of metal chloride with ethanol yields an incomplete substitution,^43^ but the reaction is driven to completion by anhydrous ammonia.^34,44^4
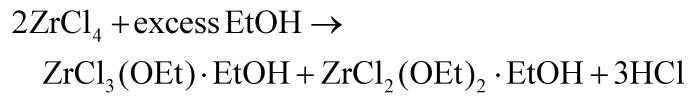

5 ZrCl_4_ + 4EtOH + 4NH_3_ → Zr(OEt)_4_ + 4NH_4_Cl

The use of sodium ethoxide is not recommended due to the formation of a heterobimetallic complex: NaZr(OEt)_5_·HOEt.^45^ The ammonia route also works well for zirconium isopropoxide, although the yields are lower. It was hypothesized that some hydrolysis occurs due to the reaction of HCl with isopropanol, generating water and isopropyl chloride.^34,44^ The effect is even worse for tertiary alcohols.^35^ Zirconium isopropoxide can be purified by recrystallization from isopropanol, yielding the Zr(OiPr)_4_·iPrOH complex. The metal alkoxides (ethoxide and isopropoxide) were also synthesized from pyridinium metal hexachloride in benzene.^35,46^6
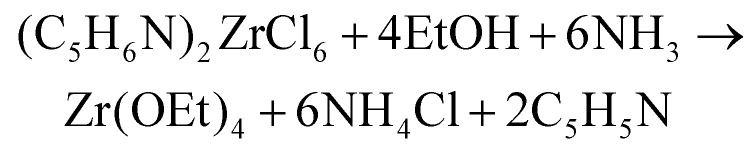


Given the convenience of purifying Zr(OiPr)_4_·iPrOH by crystallization, it is often used as starting point to synthesize other alkoxides by alcohol interchange, also called alcoholysis.^34,44,46–49^

7 Zr(OiPr)_4_·iPrOH + 4ROH → Zr(OR)_4_ + 5iPrOH

This is particularly successful when the incoming alcohol has a higher boiling point than isopropanol, *e.g.*, amyl alcohols. In case of only slight differences in boiling points of the alcohols, transesterification can be helpful.^50^ Taking the example of acetate esters:

8 Zr(OiPr)_4_·iPrOH + 4ROAc → Zr(OR)_4_ + 4iPrOAc + iPrOH

The method has produced zirconium *n*-butoxide, *sec*-butoxide and *tert*-butoxide in semi-quantitative yields.^50^ Since the isopropyl ester is more volatile, it can be fractionated out of the system. Especially for zirconium and hafnium *tert*-butoxides, the method is superior to alcohol exchange since the latter does not proceed further than metal tri-*tert*-butoxide mono-ethoxide.

Finally, one can readily generate alkoxides from metal amides, such as dialkylamides.^41^

9 Zr(NEt_2_)_4_ + 4ROH → Zr(OR)_4_ + 4HNEt_2_

This method has several advantages. (1) It is a convenient and versatile method to synthesize different metal alkoxides simply by adding the corresponding alcohol and the by-product is volatile. (2) It prevents hydrolysis which can occur when starting from the metal chloride. Although the addition of excess base (*i.e.* starting from the pyridinium hexachlorozirconate) slows the hydrolysis, in the case of *tert*-butoxide synthesis it still has a considerable impact on the yield.^35^

To prepare Zr(OiPr)_4_·iPrOH, we choose the ammonia route from ZrCl_4_ for several reasons. (1) ZrCl_4_ can be bought pure, in high quantities, and is an economical precursor. (2) Ammonia can be added to the mixture in either gas-form or as a 2 M solution in isopropanol. (3) The excess of ammonia is easily removed due to its volatility. (4) The ammonium chloride by-product precipitates from the reaction mixture and is easily removed by filtration.^34,35,44,46^ The reaction according to eqn (9) is less convenient also because the amine coordinates to zirconium isopropoxide.^36^ Zr(OiPr)_4_·iPrOH is poorly soluble in pure isopropanol and thus requires large volume of solvent for recrystallization. Fortunately, recrystallization from a toluene/isopropanol (3 : 1) mixture is much more convenient.^31^

**Fig. 1:**
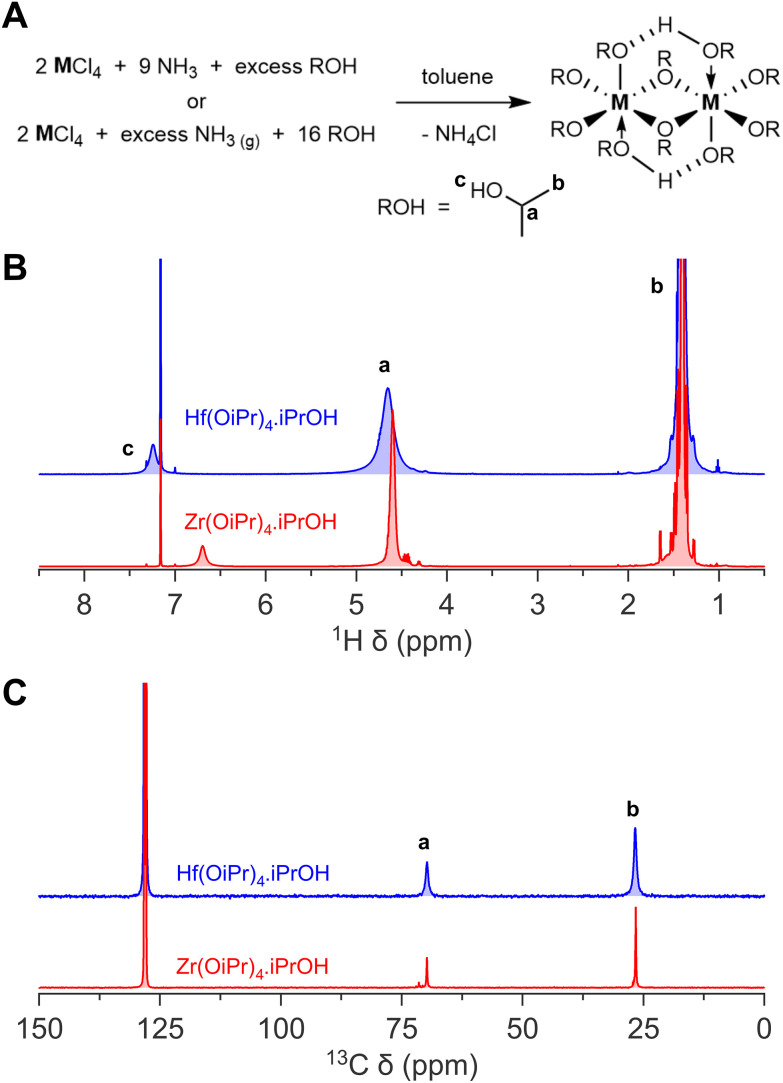
The synthesis of zirconium(iv) and hafnium(iv) isopropoxide isopropanol complex. (A) General reaction scheme of both the method making use of ammonia stock solution in isopropanol, and gaseous ammonia, (B) ^1^H NMR, and (C) ^13^C NMR of zirconium(iv) and hafnium(iv) isopropoxide isopropanol complex in C_6_D_6_.

We thus synthesized Zr(OiPr)_4_·iPrOH and Hf(OiPr)_4_·iPrOH by dispersing the metal chloride in toluene, and slowly adding a 2 M ammonia solution in isopropanol, or by bubbling gaseous ammonia through the reaction mixture while slowly adding isopropanol. We chose specifically not to start from the pyridinium hexachlorozirconate due to the high solubility of the pyridinium chloride by-product in toluene. It would require more recrystallization steps to obtain a pure final product. After filtering off the ammonium chloride and a recrystallization step, we obtain the pure white crystals. Their NMR and powder XRD spectra as shown in Fig. 1 and Fig. S2† respectively.

There are some practical remarks with either synthesis method. When the ammonia solution in isopropanol is used, the final reaction mixture has a larger volume and contains a larger fraction of isopropanol. This causes some of the formed ammonium chloride to dissolve in the reaction mixture. Consequently, a second (air- and moisture-free) filtration is required, increasing the overall workload. This is not required with the gaseous ammonia method, resulting in an overall faster and less complex process. The gaseous ammonia method is also more versatile since only a few alcoholic ammonia solutions are commercially available. Ammonia solutions in, *e.g.*, 2-butanol, are not available, limiting the possible metal alkoxides. Ammonia solutions for general use have a much lower concentration, *e.g.*, 0.5 M in dioxane, compared to 2 M in isopropanol. Gaseous ammonia avoids all these limitations but ammonia is a toxic gas, delivered in a container under pressure, and requires more experience to handle. Since both methods are similar in quality and cost, both are presented in this paper. While both the zirconium and hafnium compounds are commercially available, their appearance varies from the desired white crystals to yellow-brown powder. It was already earlier advised to recrystallize the commercial precursors.^31^

**Fig. 2:**
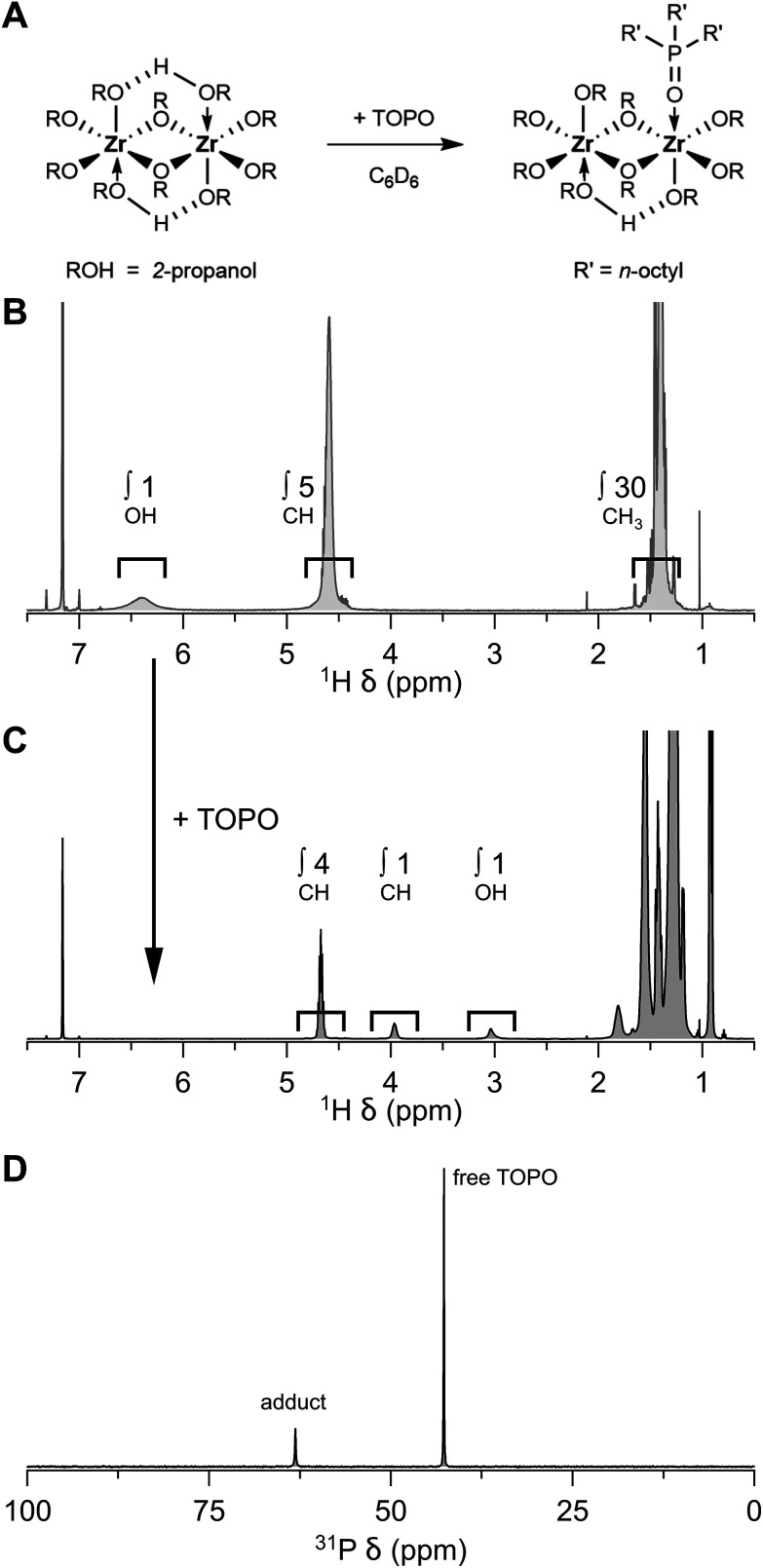
Exchange reaction of zirconium isopropoxide isopropanol complex with the more Lewis base TOPO. (A) General reaction scheme, (B) ^1^H NMR of the as-synthesized zirconium(iv) isopropoxide isopropanol complex where the ratio between the three resonances (6.5, 4.5 and 1.5 ppm) equals to 1 : 5 : 30 (= 4 isopropoxides and 1 isopropanol), (C) to which an excess of TOPO is added where the ratio between the three resonances (4.5, 4.0 and 3.0 ppm) equals to 4 : 1 : 1 (confirms the correct stoichiometry of 4 isopropoxides and 1 isopropanol), and (D) ^31^P NMR of the mixture in C_6_D_6_.

One can conveniently check the quality of the isopropoxide isopropanol complex (4 to 1 ratio) by adding tri-*n*-octylphosphine oxide (TOPO). TOPO replaces the coordinated isopropanol and one can assess the stoichiometry, *i.e.*, the ratio between isopropanol and isopropoxide, by ^1^H NMR, see Fig. 2. While the CH resonance of bound isopropoxide appears at 4.67 ppm, the CH resonance of free isopropanol appears at 3.97 ppm (in C_6_D_6_). In ^31^P NMR, the purity of the compound can be further assessed, see Fig. 2D. In case of a pure product, a single TOPO adduct is observed at 63 ppm (next to the resonance of free TOPO at 42 ppm).^18^ When the chloride exchange is incomplete, other TOPO complexes will be observed, *e.g.*, ZrCl(OR)_3_(TOPO)_2_ at 58 ppm.^28^

**Fig. 3:**
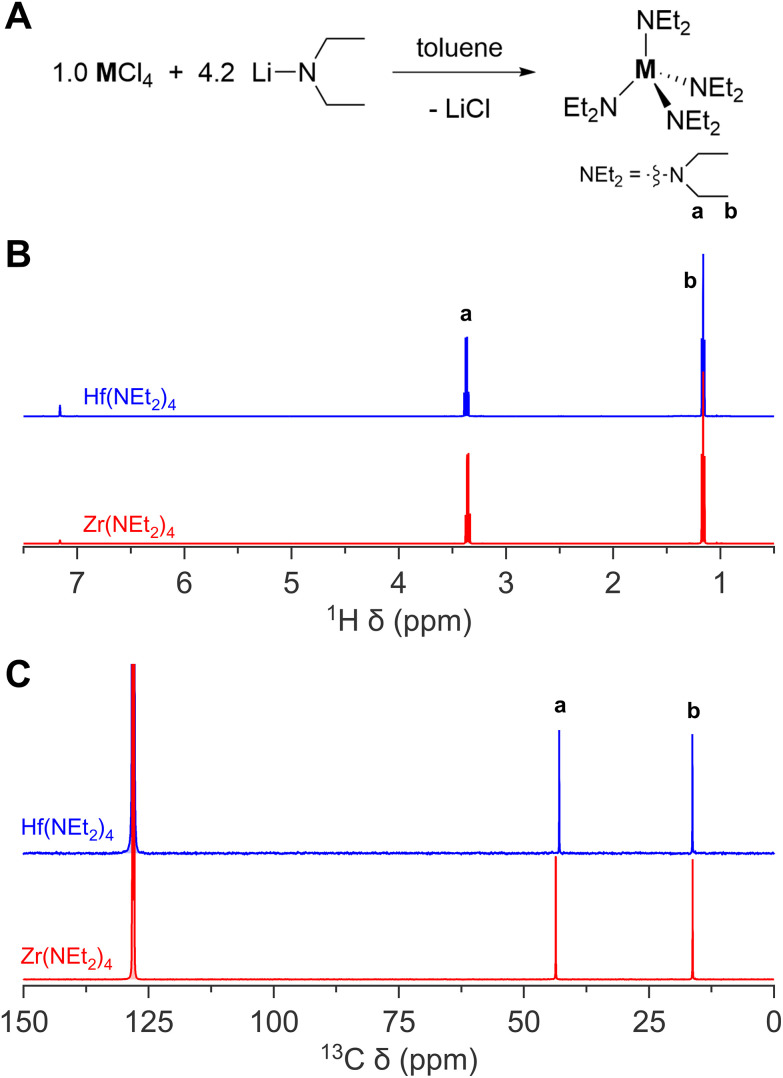
The synthesis of zirconium(iv) and hafnium(iv) diethylamido complex. (A) General reaction scheme, (B) ^1^H NMR, and (C) ^13^C NMR of zirconium(iv) and hafnium(iv) diethylamido complex in C_6_D_6_.

Tetrakis(diethylamido)-zirconium and -hafnium are synthesized from its metal chloride reacting with lithium diethylamide, see Fig. 3.^38^ The formation and precipitation of lithium chloride as by-product, which is easily removed by filtration, is the driving force of the reaction. The compound is conveniently purified by vacuum distillation and the NMR spectra as shown in Fig. 3.

**Fig. 4:**
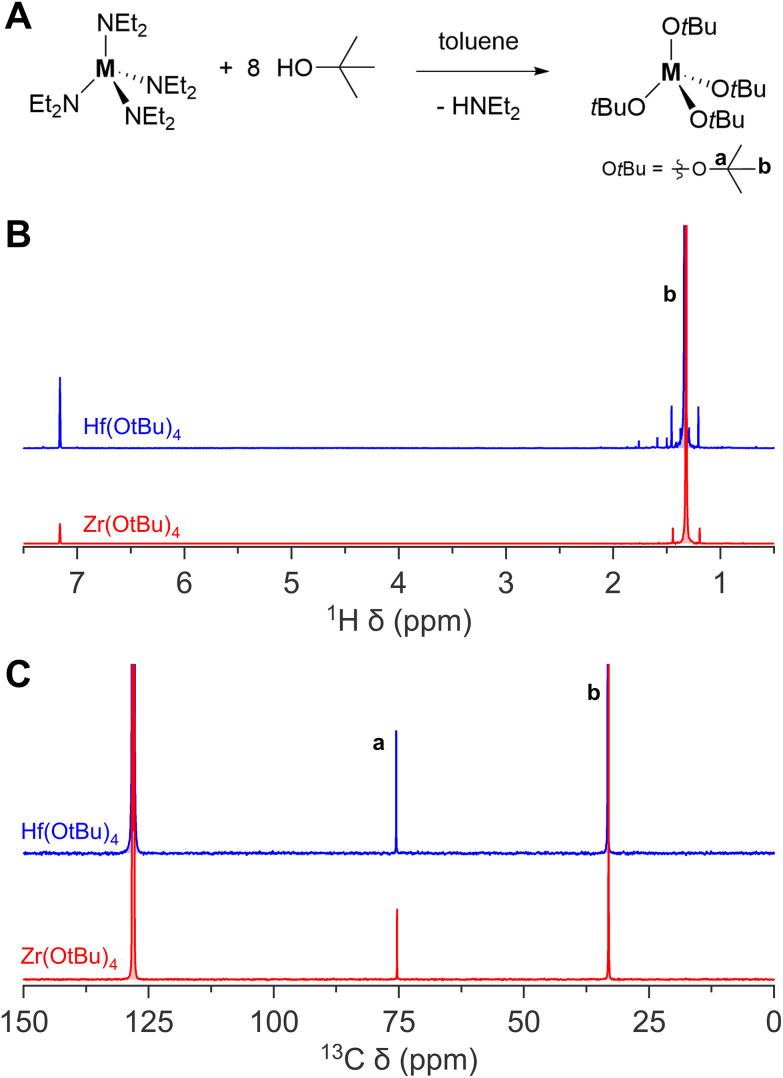
The synthesis of zirconium(iv) and hafnium(iv) *tert*-butoxide complex. (A) General reaction scheme, (B) ^1^H NMR, and (C) ^13^C NMR of zirconium(iv) and hafnium(iv) *tert*-butoxide complex in C_6_D_6_.

Zirconium and hafnium *tert*-butoxides are readily synthesized from these diethylamides upon addition of the alcohol.^41^ The products are vacuum distilled to purity. The NMR spectra are shown in Fig. 4. Although zirconium and hafnium *tert*-butoxide are commercially available, they are not delivered as completely colourless and transparent liquids. In some cases, the solution is yellowish and/or a little turbid. If one chooses to buy these products commercially, one is advised to purify them by vacuum distillation before use.

**Fig. 5:**
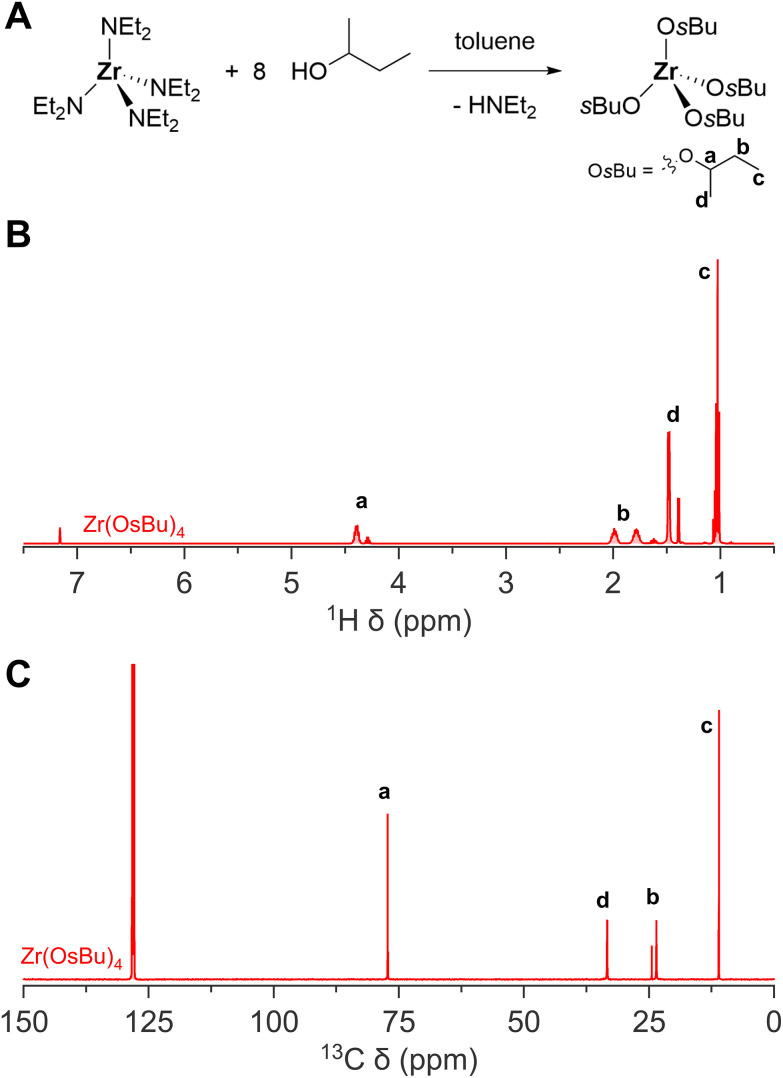
The synthesis of zirconium(iv) *sec*-butoxide. (A) General reaction scheme, (B) ^1^H NMR, and (C) ^13^C NMR of zirconium(iv) *sec*-butoxide in C_6_D_6_.

The zirconium and hafnium diethylamides are extremely versatile and can be used to synthesize a library of other alkoxides that are not commercially available. For example, we synthesized zirconium *sec*-butoxide from anhydrous 2-butanol and zirconium diethylamide, see Fig. 5. Previous attempts to synthesize zirconium *sec*-butoxide *via* the gaseous ammonia method faced considerable hydrolysis, resulting in low yield (24%).^34,35^ When starting from zirconium diethylamide, this is not an issue, and we obtain good yields (57% after vacuum destillation and isolation). Alternatively, one could also synthesize zirconium *sec*-butoxide from zirconium isopropoxide isorpropanol complex *via* alcohol exchange and fractional destillation,^34^ but we find the method from zirconium diethylamide more convenient.

**Fig. 6:**
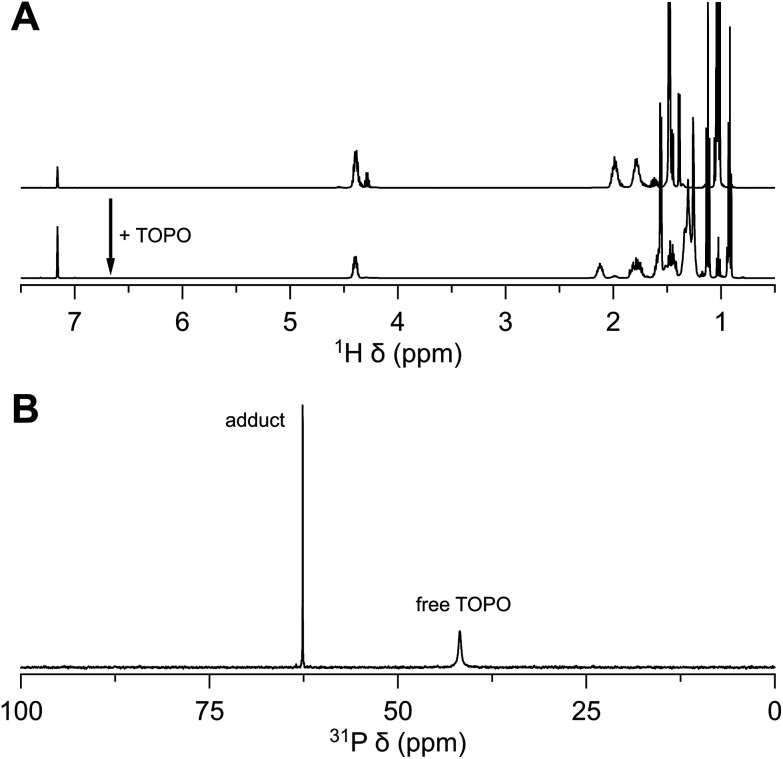
Interaction of zirconium *sec*-butoxide with TOPO. (A) ^1^H NMR of the as-synthesized zirconium(iv) *sec*-butoxide to which TOPO is added, and (B) ^31^P NMR of the mixture in C_6_D_6_.

While zirconium *sec*-butoxide can, in principle be recrystallized from 2-butanol as the solvent adduct, the coordinated alcohol is easily lost upon drying under vacuum at room temperature.^34^ Therefore, one can safely assume that the product after vacuum destillation (at 170 °C) is free from coordinated 2-butanol. Nevertheless, the ^1^H and ^13^C NMR spectra feature a second set of *sec*-butoxide resonances, indicating that the *sec*-butoxides are not all in an identical chemical environment and there is slow exchange between the different environments. This could be due to an association equilibrium. To exclude the possibility of a solvent adduct, as in the case of zirconium isopropoxide isopropanol complex, we added TOPO. Upon addition of TOPO, only one set of *sec*-butoxide resonances are observed, pointing to a more uniform chemical environment of all the *sec*-butoxide ligands. We infer that the association equilibrium was suppressed in favor of coordination by TOPO. From ^31^P NMR, a similar shift of TOPO (63 ppm) is observed as its interaction with zirconium isopropoxide complex (Fig. 6B).

## Conclusion

We identified the most convenient routes to produce zirconium and hafnium isopropoxide, *tert*-butoxide and *sec*-butoxide. We optimized, modernized, and clearly described the synthesis protocols.

## Data availability

The raw data for all the figures are openly available on Zenodo: https://doi.org/10.5281/zenodo.11486432.

## Conflicts of interest

The authors declare no competing interest.

## Supplementary Material

DT-053-D4DT01280A-s001
