# Supplementary material for: Synthesis of zirconium(iv) and hafnium(iv) isopropoxide, sec-butoxide and tert-butoxide
Source: Dalton Trans. 2024 Jun 28;53(28):11769–77. doi: 10.1039/d4dt01280a (PMC11250499; doi:10.1039/d4dt01280a)
Supplement: DT-053-D4DT01280A-s001 [file DT-053-D4DT01280A-s001.pdf]

Electronic Supplementary Material (ESI) for Dalton Transactions.
This journal is © The Royal Society of Chemistry 2024

Supporting Information: Synthesis of Zirconium(IV) and Hafnium(IV) isopropoxide, sec-butoxide, and tert-butoxide

Evert Dhaene, Carlotta Seno, and Jonathan De Roo*

Department of Chemistry, University of Basel, Mattenstrasse 22, 4058 Basel, Switzerland

E-mail: Jonathan.DeRoo@unibas.ch

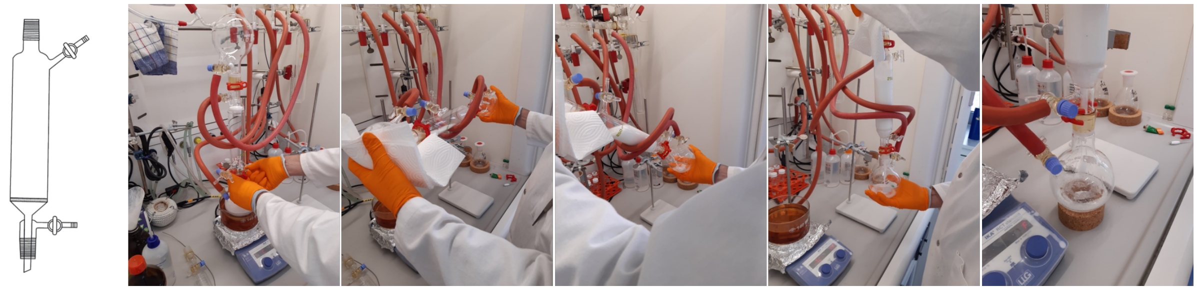

Figure S1: Schematic diagram of the reversible glass frit and pictures of the air- and moisture-free Schlenk filtration.

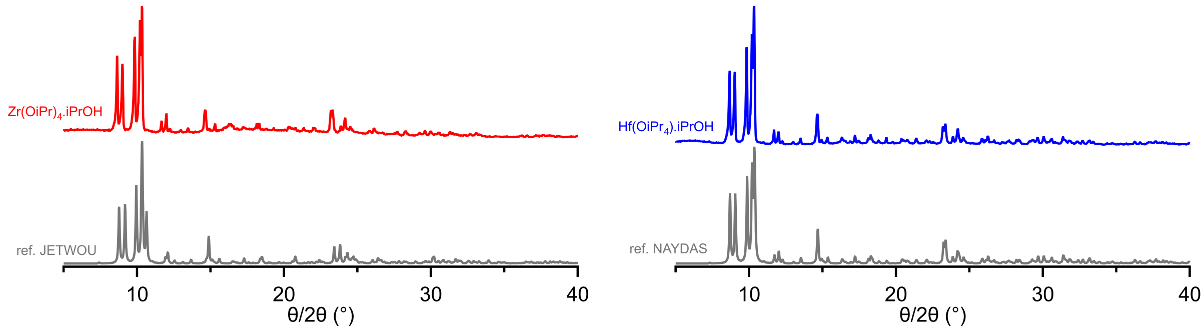

Figure S2: Powder XRD of (left) the synthesized Zr(O*i*Pr)$_4$.*i*PrOH and the calculated powder diffraction pattern from the CSD reference JETWOU, and (right) the synthesized Hf(O*i*Pr)$_4$.*i*PrOH and the calculated powder diffraction pattern from the CSD reference NAYDAS.
